# Ovarian ferroptosis induced by androgen is involved in pathogenesis of PCOS

**DOI:** 10.1093/hropen/hoae013

**Published:** 2024-02-28

**Authors:** Xinyu Li, Yunying Lin, Xiaoyue Cheng, Guangxin Yao, Jufang Yao, Shuanggang Hu, Qinling Zhu, Yuan Wang, Ying Ding, Yao Lu, Jia Qi, Hanting Zhao, Xuejiao Bian, Yanzhi Du, Kang Sun, Hugo Vankelecom, Yun Sun

**Affiliations:** Department of Reproductive Medicine, Ren Ji Hospital, Shanghai Jiao Tong University School of Medicine, Shanghai, China; Shanghai Key Laboratory for Assisted Reproduction and Reproductive Genetics, Shanghai, China; Department of Reproductive Medicine, Ren Ji Hospital, Shanghai Jiao Tong University School of Medicine, Shanghai, China; Shanghai Key Laboratory for Assisted Reproduction and Reproductive Genetics, Shanghai, China; Department of Reproductive Medicine, Ren Ji Hospital, Shanghai Jiao Tong University School of Medicine, Shanghai, China; Shanghai Key Laboratory for Assisted Reproduction and Reproductive Genetics, Shanghai, China; Department of Reproductive Medicine, Ren Ji Hospital, Shanghai Jiao Tong University School of Medicine, Shanghai, China; Shanghai Key Laboratory for Assisted Reproduction and Reproductive Genetics, Shanghai, China; Animal Laboratory, Renji Hospital, School of Medicine, Shanghai Jiao Tong University, Shanghai, China; Department of Reproductive Medicine, Ren Ji Hospital, Shanghai Jiao Tong University School of Medicine, Shanghai, China; Shanghai Key Laboratory for Assisted Reproduction and Reproductive Genetics, Shanghai, China; Department of Reproductive Medicine, Ren Ji Hospital, Shanghai Jiao Tong University School of Medicine, Shanghai, China; Shanghai Key Laboratory for Assisted Reproduction and Reproductive Genetics, Shanghai, China; Department of Reproductive Medicine, Ren Ji Hospital, Shanghai Jiao Tong University School of Medicine, Shanghai, China; Shanghai Key Laboratory for Assisted Reproduction and Reproductive Genetics, Shanghai, China; Department of Reproductive Medicine, Ren Ji Hospital, Shanghai Jiao Tong University School of Medicine, Shanghai, China; Shanghai Key Laboratory for Assisted Reproduction and Reproductive Genetics, Shanghai, China; Department of Reproductive Medicine, Ren Ji Hospital, Shanghai Jiao Tong University School of Medicine, Shanghai, China; Shanghai Key Laboratory for Assisted Reproduction and Reproductive Genetics, Shanghai, China; Department of Reproductive Medicine, Ren Ji Hospital, Shanghai Jiao Tong University School of Medicine, Shanghai, China; Shanghai Key Laboratory for Assisted Reproduction and Reproductive Genetics, Shanghai, China; Department of Reproductive Medicine, Ren Ji Hospital, Shanghai Jiao Tong University School of Medicine, Shanghai, China; Shanghai Key Laboratory for Assisted Reproduction and Reproductive Genetics, Shanghai, China; Department of Reproductive Medicine, Ren Ji Hospital, Shanghai Jiao Tong University School of Medicine, Shanghai, China; Shanghai Key Laboratory for Assisted Reproduction and Reproductive Genetics, Shanghai, China; Laboratory of Tissue Plasticity in Health and Disease, Department of Development and Regeneration, Cluster Stem Cell Biology and Embryology, Research Unit of Stem Cell Research, University of Leuven (KU Leuven), Leuven, Belgium; Department of Reproductive Medicine, Ren Ji Hospital, Shanghai Jiao Tong University School of Medicine, Shanghai, China; Shanghai Key Laboratory for Assisted Reproduction and Reproductive Genetics, Shanghai, China; Department of Reproductive Medicine, Ren Ji Hospital, Shanghai Jiao Tong University School of Medicine, Shanghai, China; Shanghai Key Laboratory for Assisted Reproduction and Reproductive Genetics, Shanghai, China; Laboratory of Tissue Plasticity in Health and Disease, Department of Development and Regeneration, Cluster Stem Cell Biology and Embryology, Research Unit of Stem Cell Research, University of Leuven (KU Leuven), Leuven, Belgium; Department of Reproductive Medicine, Ren Ji Hospital, Shanghai Jiao Tong University School of Medicine, Shanghai, China; Shanghai Key Laboratory for Assisted Reproduction and Reproductive Genetics, Shanghai, China

**Keywords:** PCOS, ferroptosis, androgen, nuclear receptor coactivator 4, ovarian dysfunction

## Abstract

**STUDY QUESTION:**

Does ovarian ferroptosis play an active role in the development of polycystic ovary syndrome (PCOS)?

**SUMMARY ANSWER:**

Increased ovarian ferroptosis was present in PCOS ovaries and the inhibition of ferroptosis with ferrostatin-1 (Fer-1) ameliorated polycystic ovary morphology and anovulation.

**WHAT IS KNOWN ALREADY:**

Programmed cell death plays a fundamental role in ovarian follicle development. However, the types and mechanisms of cell death involved in the ovary are yet to be elucidated. Ferroptosis is a recently discovered iron-dependent programmed cell death. Impaired iron metabolism and cell death have been observed in women with PCOS, the main cause of anovulatory infertility. Additionally, previous studies reported that an abnormal expression of noncoding RNA may promote ferroptosis in immortalized ovarian granulosa cell lines. However, little is known about whether ovarian ferroptosis is increased in PCOS, and there is insufficient direct evidence for a role of ferroptosis in PCOS, and the underlying mechanism. Moreover, the effect of the inhibition of ferroptosis with Fer-1 in PCOS remains unclear.

**STUDY DESIGN, SIZE, DURATION:**

Ferroptosis was evaluated in human granulosa cells (hGCs) from non-PCOS (n = 6–16) and PCOS (n = 7–18) patients. The experimental study was completed *in vitro* using primary hGCs from women undergoing IVF. Improvements in PCOS indicators following ferroptosis inhibition with Fer-1 were investigated in a dehydroepiandrosterone (DHEA)-induced PCOS rat model (n = 8 per group).

**PARTICIPANTS/MATERIALS, SETTING, METHODS:**

Ovarian ferroptosis was evaluated in the following ways: by detecting iron concentrations via ELISA and fluorescent probes; measuring malondialdehyde (MDA) concentrations via ELISA; assessing ferroptosis-related protein abundance with western blotting; observing mitochondrial morphology with transmission electron microscopy; and determining cell viability. Primary hGCs were collected from women undergoing IVF. They were treated with dihydrotestosterone (DHT) for 24 h. The effect of DHT on ferroptosis was examined in the presence or absence of small interfering RNA-mediated knockdown of the putative receptor coregulator for signaling molecules. The role of ovarian ferroptosis in PCOS progression was explored *in vivo* in rats. The DHEA-induced PCOS rat model was treated with the ferroptosis inhibitor, Fer-1, and the oocytes and metaphase II oocytes were counted after ovarian stimulation. Additionally, rats were treated with the ferroptosis inducer, RSL3, to further explore the effect of ferroptosis. The concentrations of testosterone, FSH, and LH were assessed.

**MAIN RESULTS AND THE ROLE OF CHANCE:**

Increased ferroptosis was detected in the ovaries of patients with PCOS and in rats with DHEA-induced PCOS. Increased concentrations of Fe^2+^ (*P *<* *0.05) and MDA (*P *<* *0.05), and upregulated nuclear receptor coactivator 4 protein levels, and downregulated ferritin heavy chain 1 (FTH1) and glutathione peroxidase 4 (GPX4) proteins were observed in the hGCs in patients with PCOS and ovaries of PCOS rats (*P *<* *0.05 versus control). DHT was shown to induce ferroptosis via activation of NOCA4-dependent ferritinophagy. The inhibition of ferroptosis with Fer-1 in rats ameliorated a cluster of PCOS traits including impaired glucose tolerance, irregular estrous cycles, reproductive hormone dysfunction, hyperandrogenism, polycystic ovaries, anovulation, and oocyte quality (*P *<* *0.05). Treating rats with RSL3 resulted in polycystic ovaries and hyperandrogenism (*P *<* *0.05).

**LARGE-SCALE DATA:**

N/A.

**LIMITATIONS, REASONS FOR CAUTION:**

Although ovarian-targeted ferroptosis inhibition may be a more targeted treatment for PCOS, the underlying mechanisms in the cycle between ferroptosis and hyperandrogenism require further exploration. Additionally, since PCOS shows high heterogeneity, it is important to investigate whether ferroptosis increases are present in all patients with PCOS.

**WIDER IMPLICATIONS OF THE FINDINGS:**

Androgen-induced ovarian ferroptosis appears to play a role in the pathogenesis of PCOS, which potentially makes it a promising treatment target in PCOS.

**STUDY FUNDING/COMPETING INTEREST(S):**

This study was supported by the National Key R&D Program of China (2023YFC2705500, 2023YFC2705505, 2019YFA0802604), National Natural Science Foundation of China (No. 82130046, 82320108009, 82101708, 82101747, and 82001517), Shanghai leading talent program, Innovative research team of high-level local universities in Shanghai (No. SHSMU-ZLCX20210201, No. SSMU-ZLCX20180401), Shanghai Jiaotong University School of Medicine, Affiliated Renji Hospital Clinical Research Innovation Cultivation Fund Program (RJPY-DZX-003) and Shanghai Municipal Education Commission—Gaofeng Clinical Medicine Grant Support (No. 20161413), Shanghai’s Top Priority Research Center Construction Project (2023ZZ02002), and Three-Year Action Plan for Strengthening the Construction of the Public Health System in Shanghai (GWVI-11.1-36). The authors report no competing interests.

WHAT DOES THIS MEAN FOR PATIENTS? Polycystic ovary syndrome (PCOS) is the most common hormonal disease in women of reproductive age, accounting for 80% of infertility caused by lack of ovulation. However, the origins and development of PCOS are still poorly understood. Ferroptosis is a recently discovered iron-dependent form of cell death, but whether it is involved in ovarian dysfunction (including egg development and ovulation) and the progression of PCOS is unknown. If ferroptosis is involved in PCOS, it could be a potential therapeutic target in PCOS. Our research discovered higher levels of ferroptosis in ovarian granulosa cells (cells critical for ovarian function and egg development) from patients with PCOS, and in the ovaries of an experimental model of PCOS. We then showed that an inhibitor of ferroptosis improved the ovarian and insulin resistance of PCOS, suggesting a possible treatment option for women with PCOS. Additionally, we proposed that hyperandrogenism (a medical condition characterized by high levels of androgens, with symptoms including acne, scalp hair loss, increased body or facial hair, and disrupted menstruation) and ovarian ferroptosis form a self-perpetuating cycle, which aggravates the problem—this finding provides another new insight into the treatment of patients with PCOS. To the best of our knowledge, this is the first study to reveal the vital role of ovarian ferroptosis in PCOS progression and the possible therapeutic effects of a ferroptosis inhibitor.

## Introduction

Polycystic ovary syndrome (PCOS) is the most common endocrine disorder in women of childbearing age and the primary cause of anovulation infertility, affecting 5–18% of women worldwide (Hamilton-Fairley and Taylor, 2003, Yildiz *et al.*, 2012). Women with PCOS are characterized by disrupted menstrual periods, hyperandrogenism, metabolic disorders, and polycystic ovaries caused by the presence of numerous arrested follicles (Norman *et al.*, 2007). PCOS, which is characterized by impaired folliculogenesis and ovulation disorders, accounts for 80% of anovulatory infertility (The Thessaloniki ESHRE/ASRM-Sponsored PCOS Consensus Workshop Group, 2008). Apart from its negative impact on female fecundity, PCOS-associated ovarian dysfunction also increases the risk of endometrial carcinoma (Shao *et al.*, 2014). Therefore, it is important to elucidate the mechanism(s) of ovarian dysfunction in PCOS and explore the potential therapeutic options.

PCOS-related ovarian dysfunction is at least partly caused by impaired and increased programmed cell death (Wang *et al.*, 2020; Zhang *et al.*, 2020a), which was found to be involved in follicular development and atresia (Matsuda *et al.*, 2012; Yadav *et al.*, 2018; Chaudhary *et al.*, 2019; Zhou *et al.*, 2019). As there are several kinds of programmed cell death, including apoptosis, necrosis, and pyroptosis, it is important to clarify the types and mechanisms of cell death involved in the granulosa cells (GCs) of PCOS. Ferroptosis, a newly discovered form of programmed cell death, is characterized by iron-dependent lipid peroxidation and increased cellular reactive oxygen species (ROS) (Dixon *et al.*, 2012; Xie *et al.*, 2016). The trigger for ferroptosis is excess cellular ferrous iron (Fe^2+^), and the underlying mechanism of this anomaly is a reduction in the amount of ferritin heavy chain 1 (FTH1), where iron is stored. According to previous studies, ferroptosis is also known as ferritinophagy, since Gao *et al.* (2016) found that autophagy is involved in the process of ferroptosis. Nuclear receptor coactivator 4 (NCOA4) is the cargo receptor of ferritinophagy (Hu *et al.*, 2004; Kollara and Brown, 2012). NCOA4 traffics the FTH1 to autophagosomes, resulting in the accumulation of Fe^2+^ and consequent ferroptotic cell death (Hou *et al.*, 2016). Although previous studies have demonstrated the presence of impaired iron metabolism in patients with PCOS (Adamska *et al.*, 2019; Bannigida *et al.*, 2020), and while noncoding RNA promotes ferroptosis in immortalized ovarian granulosa cell lines (Zhang *et al.*, 2021; Tan *et al.*, 2022), still there is no direct association between PCOS and ferroptosis in patients or animal models that have been studied.

NCOA4, which is also known as androgen receptor (AR)-associated coregulator 70 (ARA70), was the first identified AR coregulator and it was found to potentiate AR transcriptional activity (Hu *et al.*, 2004; Kollara and Brown, 2012). Since hyperandrogenism is recognized as one of the core features of PCOS pathophysiology (Legro *et al.*, 1998), we hypothesized that androgen overexposure induces ferroptosis in the ovary, and this may play an important role in the development of PCOS.

In the present study, the effects and mechanisms of ovarian ferroptosis in PCOS were investigated. We demonstrated that the ferroptosis occurred aberrantly in the ovaries of patients with PCOS and in rats with dehydroepiandrosterone (DHEA)-induced PCOS. Treatment with a ferroptosis inhibitor, Ferrostatin-1 (Fer-1), ameliorated a cluster of PCOS traits in the rat model, while treatment with a ferroptosis activator induced the PCOS-like changes in experimental rats. Additionally, we reported that ferroptosis in ovarian GCs was mediated by dihydrotestosterone (DHT)-induced NCOA4-dependent ferritinophagy. Taken together, our study verified a crucial role for ovarian ferroptosis in the progression of PCOS and suggested that ovarian ferroptosis is a potential therapeutic target for treatment of PCOS.

## Materials and methods

### Cell collection and culture

Ovarian GCs were collected from patients with PCOS and non-PCOS controls, following the 2003 Rotterdam criteria (Dumesic *et al.*, 2015), who underwent IVF or ICSI at the Centre for Reproductive Medicine, Ren Ji Hospital, Shanghai Jiao Tong University School of Medicine. All patients recruited were treated with a standard GnRH antagonist stimulation protocol followed by an hCG trigger, GnRH agonist, or a dual trigger to induce ovulation. Baseline serum hormones were determined using chemiluminescence assay kits (DxI 800; Beckman, Access Health Co, Brea, CA, USA). All experimental procedures were approved by the Ethics Committee of Ren Ji Hospital, Shanghai Jiao Tong University School of Medicine (RA-2021-251). After follicular fluid samples were collected, we used Ficoll-Paque^TM^ PLUS (GE HealthCare Bio-Sciences, Uppsala, Sweden) to extract GCs (Zhu *et al.*, 2016). The GCs extracted from the non-PCOS and PCOS patients were collected to detect the ferroptosis level in individual patients. Additionally, to explore the effect of DHT on GCs, the GCs extracted from additional non-PCOS patients were pooled and cultured with DMEM/Ham’s F12 medium (DMEM/F12) (Gibco, Grand Island, NY, USA) supplemented with 10% fetal bovine serum (FBS) (Gibco) at 5% CO_2_ and 37°C for 2 days. Then the GCs were cultured in an FBS-free medium with DHT for 24 h.

### siRNA transfection with electroporation

GCs were transfected after isolation from the follicular fluid with 50 nM siRNA against NOCA4 (5′-GACCUUAUUUAUCAGCUUA-3′) or a negative control (5′-UUCUCCGAACGUGUCACGUTT-3′) (GenePharma, Shanghai, China) with Opti-MEM (Life Technologies Inc.) using an electroporator (Nepa Gene, Chiba, Japan) at 167 V for 5 ms according to Lu et al. (2021). The GCs were then cultured with 10% FBS and antibiotics for 2 days before treatment.

### Cell viability measurement

After treating GCs with DHT (Meilunbio, Dalian, China) or inhibitor Fer-1 (CSN12654, CSNpharm, Chicago, IL, USA), GC viability was measured using a Cell Counting kit-8 (CCK-8) (Dojindo Laboratories, Kumamoto, Japan).

### Fe^2+^ staining of GCs

Thereafter, the GCs from the non-PCOS and PCOS patients were collected separately and cultured for 24 h. For DHT treatment, pooled GCs were collected and then treated with DHT for 24 h. The intracellular fluorescent probes FerroOrange (Dojindo Laboratories, Kumamoto, Japan) were used to detect the cellular levels of Fe^2+^ according to the manufacturer’s instructions. The Dragonfly 200 fluorescence confocal microscope (Andor, Oxford, UK) was used for acquisition. The fluorescence intensity of each cell was pictured in at least five or more random views and analyzed with the Image J software (National Institutes of Health, Bethesda, MD, USA).

### Measuring Fe^2+^ and malondialdehyde concentrations

The Fe^2+^ concentrations in GCs from both non-PCOS and PCOS patients and in ovaries from rats were detected using an Iron Assay Kit (Abcam, Cambridge, UK) as per the manufacturer’s instructions. The malondialdehyde (MDA) concentrations in GCs were detected using the Lipid Peroxidation MDA Assay Kit (Beyotime, Shanghai, China).

### Transmission electron microscopy

After fixing with 2% glutaraldehyde/0.1 M phosphate buffer for 48 h, GCs collected from both non-PCOS and PCOS patients and from rat ovaries were dehydrated using ethanol in a graded series manner. Then, transmission electron microscopy (TEM) (Hitachi, Tokyo, Japan) was used to observe the fixed cells and ovarian tissues.

### Western blotting

The GCs and rat ovarian tissue were lysed in RIPA lysis buffer (Shenggong, Shanghai, China) containing a protease inhibitor cocktail (Roche, Basel, Switzerland) on ice, after which the supernatant was collected. After being quantified using the Bradford assay (Beyotime), 30 μg of proteins were loaded onto 10–12.5% sodium dodecyl sulfate polyacrylamide gels. After being transferred to nitrocellulose membranes and blocked with 5% non-fat milk for 1 h at room temperature, membranes were incubated with primary antibody and secondary antibody. The peroxidase activity of bands was detected using the chemiluminescent detection kit (Millipore Sigma, Burlington, MA, USA). The primary and secondary antibodies used were: NCOA4 (raised in rabbit, 1:500; Abcam), FTH1 (raised in rabbit, 1:1000; Abcam), GPX4 (raised in rabbit, 1:1000; Abcam), and GAPDH (raised in mouse, 1:10000; Proteintech, Wuhan, China).

### Establishment of the rat model

All Sprague-Dawley (SD) rats were purchased from the Jiesijie Animal Laboratory (Shanghai, China). Animal experiments were approved by the Animal Care Committee of the Ren Ji Hospital, Shanghai Jiao Tong University School of Medicine. To establish the PCOS rat model, 3-week-old female SD rats were injected daily (s.c.) with DHEA (6 mg/100 g body weight (BW)) (Langchem, Shanghai, China) dissolved in 0.2 ml PBS for 20 consecutive days, after 1 week of adaptation, according to Li *et al.* (2021b). Meanwhile, the rats in the control group were injected with an equivalent volume of PBS. To explore the role of ferroptosis in PCOS, 3-week-old female rats were divided into three groups. The PCOS rats from the PCOS + Fer-1 group were treated with Fer-1 (CSN12654, CSNpharm, Chicago, IL, USA) (5 mg/kg, BW) or equivalent amounts of PBS by injection (i.p.) once every 2 days for 20 days. Rats from the other two groups were treated with equivalent amounts of PBS. All rats underwent estrous cycle tests for 8 days before model establishment. After model establishment, eight rats from each group were euthanized and the remaining rats underwent ovarian stimulation. To explore the effect of ferroptosis on the ovary, 3-week-old female SD rats were treated with RSL3 (10 μM, i.p.) (MCE, Shanghai, China) once every 2 days for a week. Rats from the control group were injected with an equivalent volume of PBS. The rates were then euthanized and the serum and ovary tissues were collected.

### Ovarian stimulation and oocyte retrieval

Rats from each experimental group underwent treatment with pregnant mare’s serum gonadotrophin (300 IU/kg) followed by hCG (300 IU/kg) (Ningbo Sansheng Pharmaceutical Co., Ltd, Zhejiang, China) 48 h later to induce superovulation. At 16 h after ovulation, we gathered cumulus–oocyte complexes in oviductal ampullae and then used 0.1% hyaluronidase (Sigma, St Louis, MO, USA) to digest and extract the oocytes. We observed the oocytes from different angles to determine whether the oocytes were at metaphase II (MII) stage or not. The retrieved oocytes and MII oocytes were counted and photographed under a microscope (Zeiss, Oberkochen, Germany).

### Glucose tolerance tests

Rats from each group were injected with D-glucose (2.0g/kg BW) i.p. after a 16 h fast (from 5 p.m. to 9 a.m.). Then, glucose levels were measured at 0, 15, 30, 60, 90, and 120 min using an Accu-Chek glucose monitor (Roche, Basel, Switzerland).

### Blood analyses

Blood samples collected from rats were centrifuged at 845*g* for 15 min at 4°C to collect serum. The serum levels of LH (Enzo Life Sciences, Farmingdale, NY, USA), FSH (Sango Biotech, Shanghai, China), and testosterone (R&D Systems, Minneapolis, ME, USA) were detected using ELISA kits. All the procedures were performed according to the manufacturers’ protocols.

### Histology and immunohistochemistry

Left or right ovaries, randomly, were fixed with 4% paraformaldehyde and then embedded in paraffin. Ovaries were serially sectioned at 5 μm, and every fifth section of the ovary was stained with hematoxylin and eosin, and images were taken using a microscope (Zeiss).

### Classification and follicle counts

Follicles at different stages of development (early antral, antral, and corpus luteum (CL)) were classified as previously described (Myers *et al.*, 2004). Follicles in every fifth section were counted as previously described (Myers *et al.*, 2004).

### Statistical analysis

All data were presented as the mean ± SEM. SPSS version 16.0 software (SPSS, Inc., Chicago, IL, USA) and GraphPad Prism version 7.0 statistical software (GraphPad, Inc., La Jolla, CA, USA) were used to conduct statistical analyses. To determine whether our data had a normal distribution, the Kolmogorov–Smirnov test was performed. For normally distributed data, we performed a paired or unpaired Student’s *t*-test and ANOVA, followed by the Tukey’s multiple comparison *post-hoc* test. For the data that were not normally distributed, we performed the Kruskal–Wallis test followed by Dunn’s *post-hoc* test. Correlations between variables were determined using Pearson’s chi-square test. The threshold for statistical significance was set as *P* < 0.05.

## Results

### Elevated ferroptosis in the ovaries of patients with PCOS and the PCOS rat model

We gathered ovarian GCs from both PCOS and non-PCOS patients who received IVF or ICSI treatment in our center. The demographic features of recruited patients are presented in Supplementary Table S1. There were no statistically significant differences between the two groups in age, BMI, and basal FSH level, while LH levels were significantly increased in PCOS patients (*P *=* *0.0183 compared to controls). The LH/FSH ratio (*P *=* *0.0031) and levels of testosterone (*P *<* *0.0001) and anti-Müllerian hormone (*P *=* *0.0006) were significantly increased in patients with PCOS compared to controls. PCOS patients had a higher retrieved oocyte count than non-PCOS patients (*P *=* *0.0170), with no difference between groups in the number of MII oocytes.

**Figure 1.**
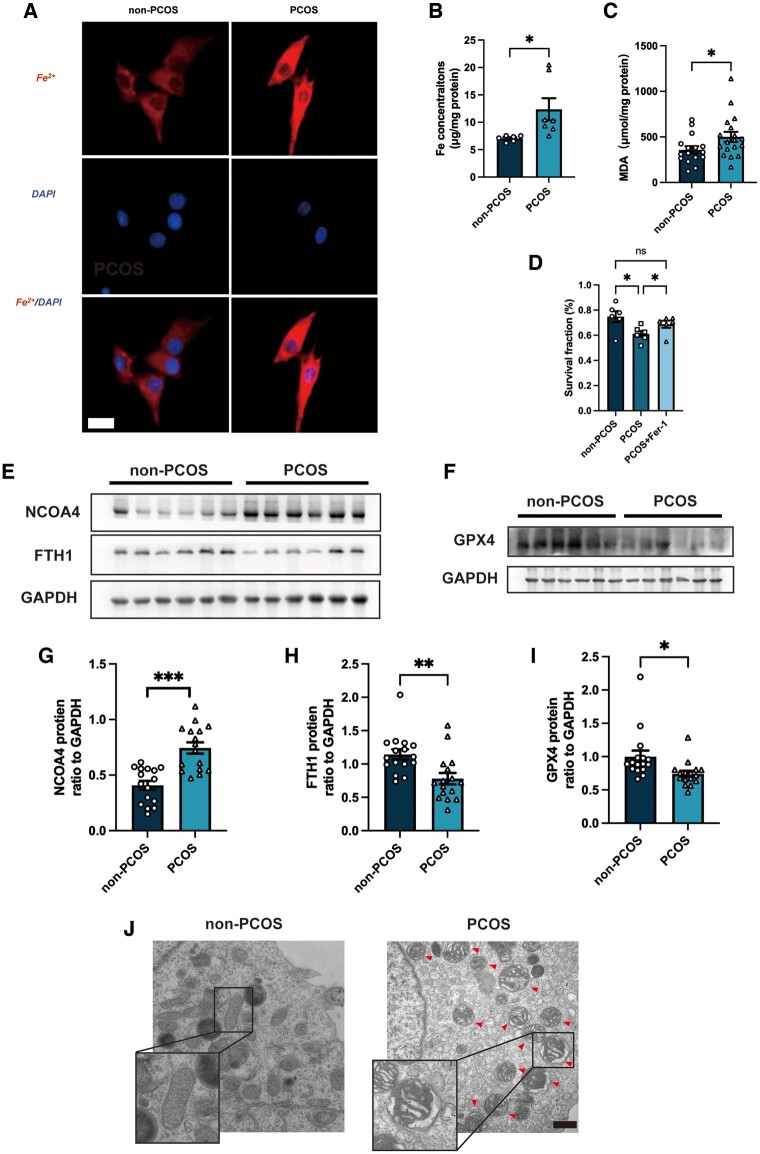
**Elevated ferroptosis in granulosa cells from patients with PCOS.** (**A**). Representative images of intracellular Fe^2+^ in GCs from PCOS patients and non-PCOS patients detected by FerroOrange (n = 3 per group). Scale bar = 20 μm. (**B** and **C**) The cellular Fe^2+^ concentration (n = 6–7 per group) and MDA concentration (n = 16–18 per group) of GCs from non-PCOS and PCOS groups were also detected. (**D**) GCs isolated from PCOS patients were treated with Fer-1 (1 μM) for 48 h and then cell viability was detected by CCK-8 (n = 6 per group). (**E**–**I**) Protein abundance of ferroptosis-related proteins NCOA4, FTH1, GPX4 in GCs from PCOS patients and non-PCOS patients (n = 16 per group), assessed by western blotting. (**J**) Representative TEM images of GCs from PCOS patients and non-PCOS patients. Scale bar = 500 nm. Red arrowheads indicate abnormal mitochondria. Data were analyzed using unpaired Student’s *t*-test and presented as mean ± SEM. *P < 0.05; **P < 0.01, ***P < 0.001, ns: no significance versus the non-PCOS group. CCK-8: cell counting kit-8; Fer-1: ferrostatin-1; FTH1: ferritin heavy chain 1; GCs: granulosa cells; GPX4: glutathione peroxidase 4; MDA: malondialdehyde; NCOA4: nuclear receptor coactivator 4; PCOS: polycystic ovary syndrome; TEM: transmission electron microscopy.

The ferroptosis levels in GCs from the two groups were measured. As increased Fe^2+^ is the trigger of ferroptosis, we measured Fe^2+^concentrations in GCs. FerroOrange staining revealed a significantly increased intracellular Fe^2+^ level in the GCs from PCOS patients compared to the non-PCOS patients (Fig. 1A and Supplementary Fig. S1A), and the same result was found using an Fe^2+^ concentration kit (Fig. 1B). Meanwhile, we detected higher MDA concentrations in GCs in the PCOS versus non-PCOS group (Fig. 1C). We observed decreased cell viability of GCs from PCOS patients compared to those from non-PCOS patients, which was reversed by treatment with the ferroptosis inhibitor Fer-1, as assessed using the CCK-8 test (Fig. 1D). However, there was no effect of Fer-1 treatment on cell viability of GCs from patients without PCOS (Supplementary Fig. S1B). Ferroptosis-related proteins were also detected to confirm the elevated ferroptosis in the GCs obtained from PCOS patients. The abundance of the ferritinophagy cargo receptor NCOA4 was increased, while the abundance of the iron storage protein FTH1 and the ferroptosis key suppressor GPX4 (Yoshida *et al.*, 2019; Yan *et al.*, 2021) was significantly decreased in PCOS patients (Fig. 1E–I). Ferroptotic cells exhibited abnormal mitochondrial morphology. Surprisingly, many more abnormal mitochondria were observed in GCs from PCOS patients (Fig. 1J and Supplementary Fig. S1C). The abnormal mitochondria appeared shorter than normal with increased membrane intensity. These findings suggested that ferroptosis was increased in ovarian GCs from PCOS patients compared to those from patients without PCOS.

**Figure 2.**
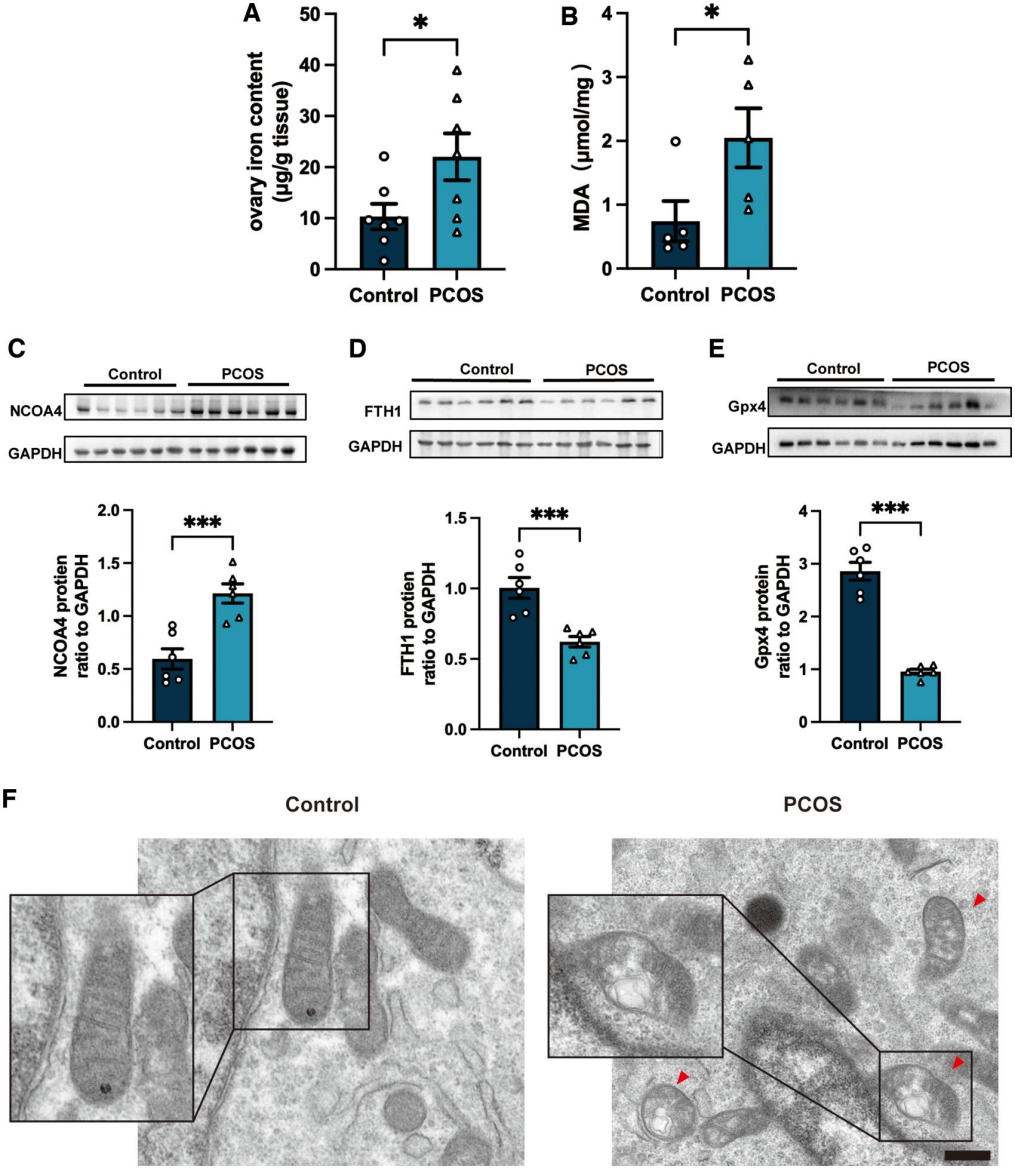
**Elevated ferroptosis in the ovaries of rats with dehydroepiandrosterone-induced PCOS.** (**A** and **B**) Fe^2+^ concentration (n = 7 per group) and MDA concentration (n = 5 per group) in the ovaries of rats from the control group and the PCOS group. (**C**–**E**) Protein abundance of ferroptosis-related proteins NCOA4, FTH1, and Gpx4 in the ovaries of rats from the two groups. (**F**) Representative TEM images of GCs in the ovaries of rats from control group and the PCOS group. Scale bar = 500 nm. Red arrowheads indicate abnormal mitochondria. Data were analyzed using unpaired Student’s *t*-test and presented as mean ± SEM. *P < 0.05; **P < 0.01, ***P < 0.001 versus the control group. Fer-1: ferrostatin-1; FTH1: ferritin heavy chain 1; GCs: granulosa cells; Gpx4: glutathione peroxidase 4; MDA: malondialdehyde; NCOA4: nuclear receptor coactivator 4; PCOS: polycystic ovary syndrome; TEM: transmission electron microscopy.

We also investigated ovarian ferroptosis in a PCOS rat model, after injecting rats with DHEA for 20 days. The successful establishment of the PCOS rat model was assessed by the estrous cycle, together with serum hormone levels and ovarian morphology, according to a previous study (Yuan *et al.*, 2016) (Supplementary Fig. S2A–F). Significantly increased Fe^2+^ concentrations with elevated MDA concentrations were also detected in ovarian tissues of PCOS rats (Fig. 2A and B). In parallel, the abundance of NCOA4 was increased in the ovaries of PCOS rats, while the abundance of Gpx4 and FTH1 was decreased in PCOS rats compared to the control rats (Fig. 2C–E). Importantly, we also observed many more abnormal mitochondria in the GCs of PCOS rats than the those of rats in the control group (Fig. 2F and Supplementary Fig. S2G). These abnormal mitochondria appeared shorter, and had disrupted cristae and compromised membrane integrity, as previously described as the characteristic morphology in ferroptosis (Badgley *et al.*, 2020) (Fig. 2G). The compromised membrane integrity reflected the injury to mitochondria and led to the death of cells (Fig. 2F). All the above implied an elevated level of ferroptosis in the ovaries of PCOS rats compared to control rats.

### Fer-1 treatment alleviated ovarian ferroptosis in the PCOS rat model

**Figure 3.**
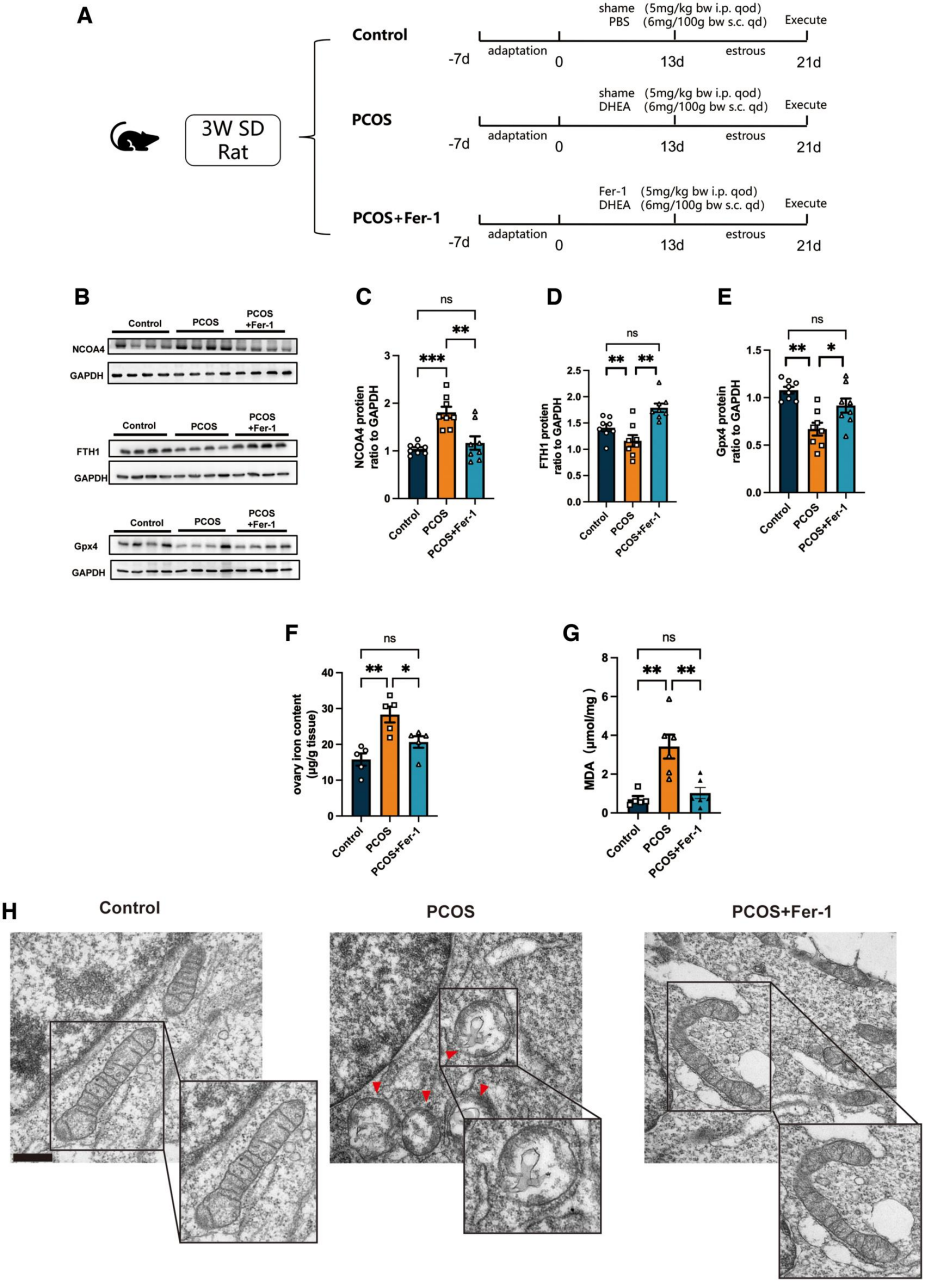
**Treatment with ferrstatin-1 alleviated ovary ferroptosis in the rat model of PCOS.** PCOS rats were treated with the ferroptosis inhibitor Fer-1 (5 mg/kg BW, i.p. qod). (**A**) Diagrammatic representation of the rats from the control group, the PCOS group, and the PCOS + Fer-1 group. (**B**–**E**) Protein abundance of ferroptosis-related proteins NCOA4, FTH1, and Gpx4 in the ovaries of rats from the three groups. (n = 8 per group). (**F** and **G**) Fe^2+^ concentration (n = 5 per group) and MDA concentration (n = 5 per group) in the ovaries of rats from the control group, the PCOS group, and the PCOS + Fer-1 group. (**H**). Representative TEM images of mitochondrial morphology in ovary GCs from the three groups. Red arrowheads indicate abnormal mitochondria. Scale bar = 500 nm. Data were analyzed using one-way ANOVA with Tukey’s multiple comparison postdoc test and presented as mean ± SEM. *P < 0.05; **P < 0.01, ns: no significance versus the control group. BW: body weight; Fer-1: ferrostatin-1; FTH1: ferritin heavy chain 1; GCs: granulosa cells; Gpx4: glutathione peroxidase 4; MDA: malondialdehyde; NCOA4: nuclear receptor coactivator 4; PCOS: polycystic ovary syndrome; qd: once a day; qod: once every other day; SD: Sprague-Dawley; TEM: transmission electron microscopy.

Based on the above findings, we then treated PCOS rats with the ferroptosis inhibitor Fer-1 (5 mg/kg BW, i.p. once every other day) to further clarify the effect of elevated ovarian ferroptosis on PCOS progression (Fig. 3A). We found that Fer-1 treatment effectively reduced the ferroptosis in ovaries obtained from PCOS rats. The abundance of NCOA4 was reduced after the Fer-1 treatment, while the decrease in Gpx4 and FTH1 levels in the ovaries of PCOS rats was reversed after Fer-1 treatment (Fig. 3B–E). The markedly increased Fe^2+^ and MDA concentrations in the ovaries of PCOS rats were also reduced after the Fer-1 treatment (Fig. 3F and G). Of note, after the Fer-1 treatment, abnormal mitochondria were significantly fewer in the GCs of PCOS rats (Supplementary Fig. S3). Using TEM, we observed that the number of shorter mitochondria with ruptured crista in GCs was significantly reduced in the PCOS+Fer-1 group (Fig. 3H). Therefore, we deduced that peritoneal injections of the ferroptosis inhibitor Fer-1 could alleviate ferroptosis in the ovary of PCOS rats.

### Fer-1 treatment improved the impaired insulin sensitivity, disrupted estrous cycle, increased LH/FSH ratio, hyperandrogenism, and abnormal ovarian morphologies in PCOS rats

**Figure 4.**
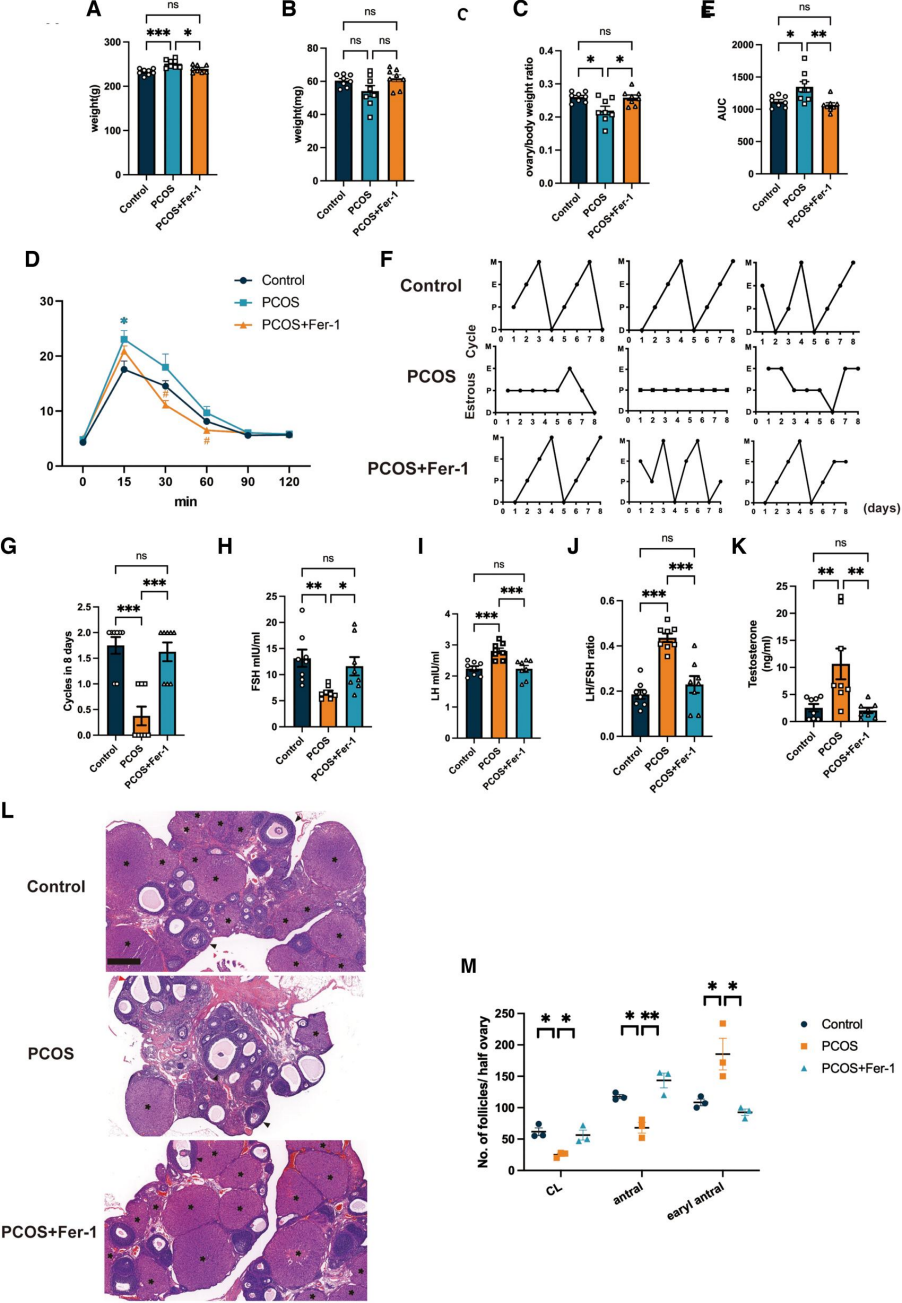
**Inhibition of ferroptosis with ferrstatin-1 reversed the increased body weight, insulin resistance, acyclicity, increased LH/FSH ratio, hyperandrogenism, and ovarian phenotypes in PCOS.** PCOS rats were treated with ferroptosis inhibitor Fer-1 (5 mg/kg BW, i.p. qod). (**A**–**C**) Body weights, ovary weights, and ovary/body weight ratio of rats from the control group, the PCOS group, and the PCOS + Fer-1 group (n = 8 per group). (**D** and **E**) Glucose tolerance test and its AUC values were assessed in rats from the control, PCOS, and PCOS + Fer-1 group (n = 8 per group). (**F** and **G**) The representative estrous cycle of rats and the number of cycles completed in 8 days in the three different experimental groups (n = 8 per group). (**H**–**K**) FSH levels, LH levels, the LH/FSH ratio, and testosterone levels of rats in the control, PCOS, and PCOS + Fer-1 group (n = 8 per group). (**L**) Representative histological section images of ovaries from the control and experimental groups. Scale bar = 500μm. Asterisks indicate corpus luteum, black arrowheads indicate antral follicles. (**M**) The number of CL, antral, and early antral follicles in the ovaries of rats from each group (n = 3 per group). Data were analyzed using one-way ANOVA with Tukey’s multiple comparison *post-hoc* test and presented as mean ± SEM. *P < 0.05; **P < 0.01; ***P < 0.001 ns: no significance versus the control group. BW: body weight; CL: corpus luteum; D: diestrus; E: estrus; Fer-1: ferrostatin-1; M: metestrus; P: proestrus; PCOS: polycystic ovary syndrome; qod: once every other day.

Since Fer-1 treatment can alleviate the increased ferroptosis in the ovaries of PCOS rats, we further explored whether the treatment would benefit PCOS traits. We found that Fer-1 treatment effectively reduced the increased BW of PCOS rats (Fig. 4A). Unlike in PCOS patients, there was no difference between ovary weight of the rats in the control group and those in the PCOS group. However, the ovary/BW ratio was decreased significantly in the PCOS group compared to the control group and reversed in the PCOS + Fer-1 group (Fig. 4B and C). A glucose tolerance test revealed impaired insulin sensitivity in the PCOS rats upon calculating the AUC of blood glucose levels, while Fer-1 treatment improved insulin sensitivity in the PCOS rats (Fig. 4D and E). Disrupted menstruation is one of the key diagnostic criteria for PCOS; we found that the irregular estrous cyclicity associated with PCOS was reversed after Fer-1 treatment (Fig. 4F and G). Additionally, the abnormal hormone profiles, including the increased LH/FSH ratio and hyperandrogenism in PCOS rats, were normalized in the PCOS + Fer-1 group (Fig. 4H–K). The polycystic ovarian morphology of PCOS rats was also alleviated in the PCOS + Fer-1 group (Fig. 4L). The CL was smaller in PCOS rats compared to the rats in the control group, indicating that fewer oocytes ovulated. Additionally, we observed fewer antral follicles and many more early antral follicles in PCOS rats than in rats of the control group, suggesting that more follicles were growth-arrested in PCOS rats than in control rats. Nevertheless, all these tendencies were reversed after the Fer-1 treatment (Fig. 4M). These suggested that Fer-1 treatment ameliorated PCOS and ferroptosis might play an important role in the progression of PCOS.

### Fer-1 treatment reversed PCOS oligo-ovulation and impaired oocyte quality

**Figure 5.**
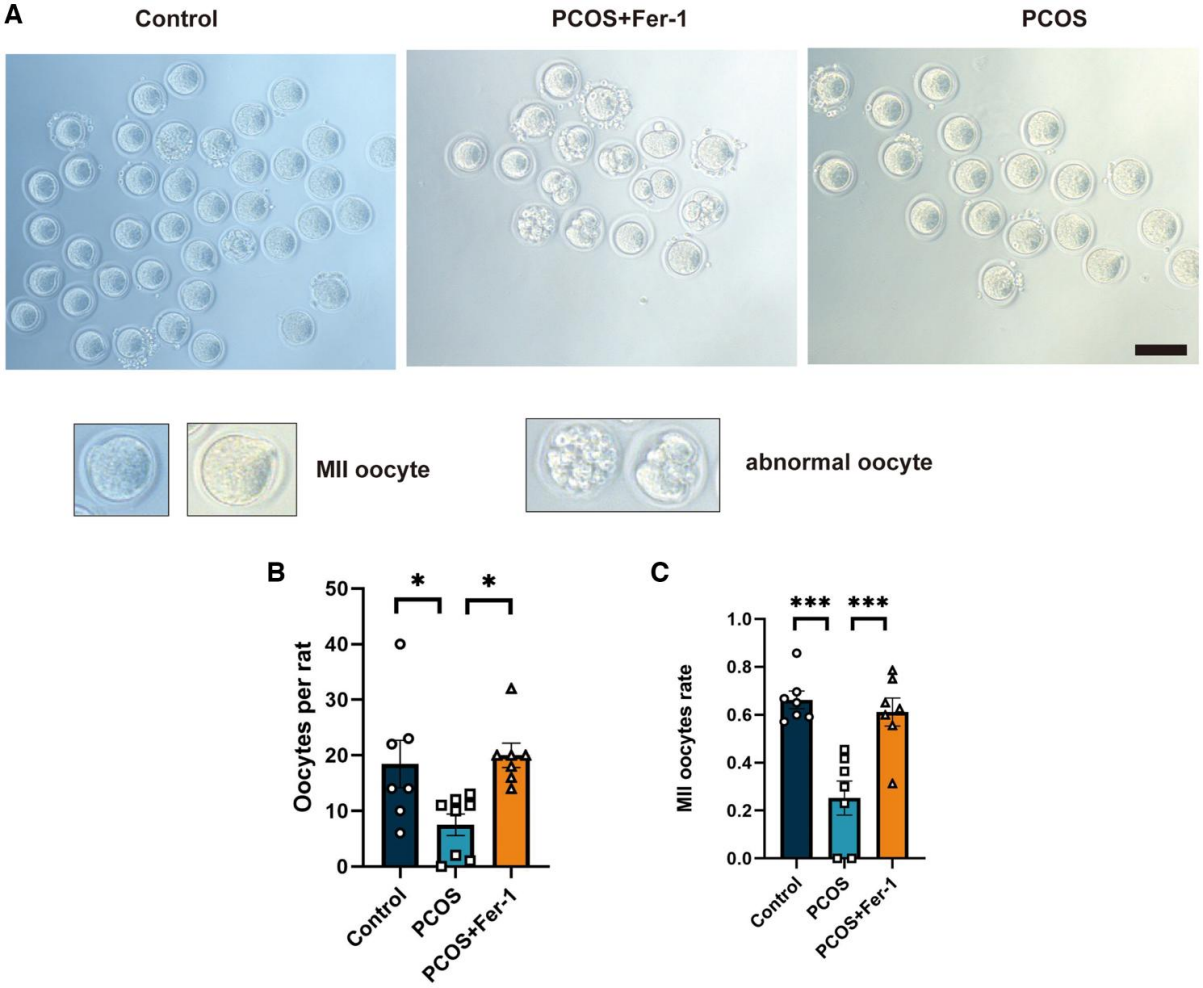
**Inhibition of ferroptosis with ferrostatin-1 reversed PCOS ovulation dysfunction and oocyte quality.** PCOS rats were treated with the ferroptosis inhibitor Fer-1 (5 mg/kg BW, i.p. qod) before inducing superovulation in the rats from the control, PCOS, and PCOS + Fer-1 group. (**A** and **B**) Representative images and number of ovulated oocytes from rats in the control, PCOS, and PCOS + Fer-1 group (n = 7–8 per group). (**C**) The MII oocytes rate of rats from the three groups (n = 7–8 per group). Scale bar =100 μm. Data were analyzed using one-way ANOVA with Tukey’s multiple comparison postdoc test and presented as mean ± SEM. *P < 0.05; ***P < 0.001 versus the control group. bw: body weight; Fer-1: ferrostatin-1; MII: metaphase II; PCOS: polycystic ovary syndrome; qod: once every other day.

PCOS was often attributed to oligo-ovulation and impaired oocyte quality since programmed cell death decides the fate of the follicle. In the present study, Fer-1 treatment improved follicular development in PCOS rats. To further confirm the effect of ovarian ferroptosis on ovarian function, super-ovulations were induced in rats of the control group, the PCOS group, and the PCOS + Fer-1 group, and the MII oocyte rate of each group was calculated. We observed more abnormal oocytes and fewer MII oocytes in PCOS rats; however, many more oocytes were collected in the oviduct and significantly higher MII oocyte rates were calculated in PCOS rats after the Fer-1 treatment (Fig. 5A–C). Hence, we further confirmed the important role of ovarian ferroptosis in the ovarian dysfunction of PCOS.

### NCOA4-dependent ferritinophagy and the GPX4 axis are involved in DHT-induced ferroptosis in ovarian GCs

**Figure 6.**
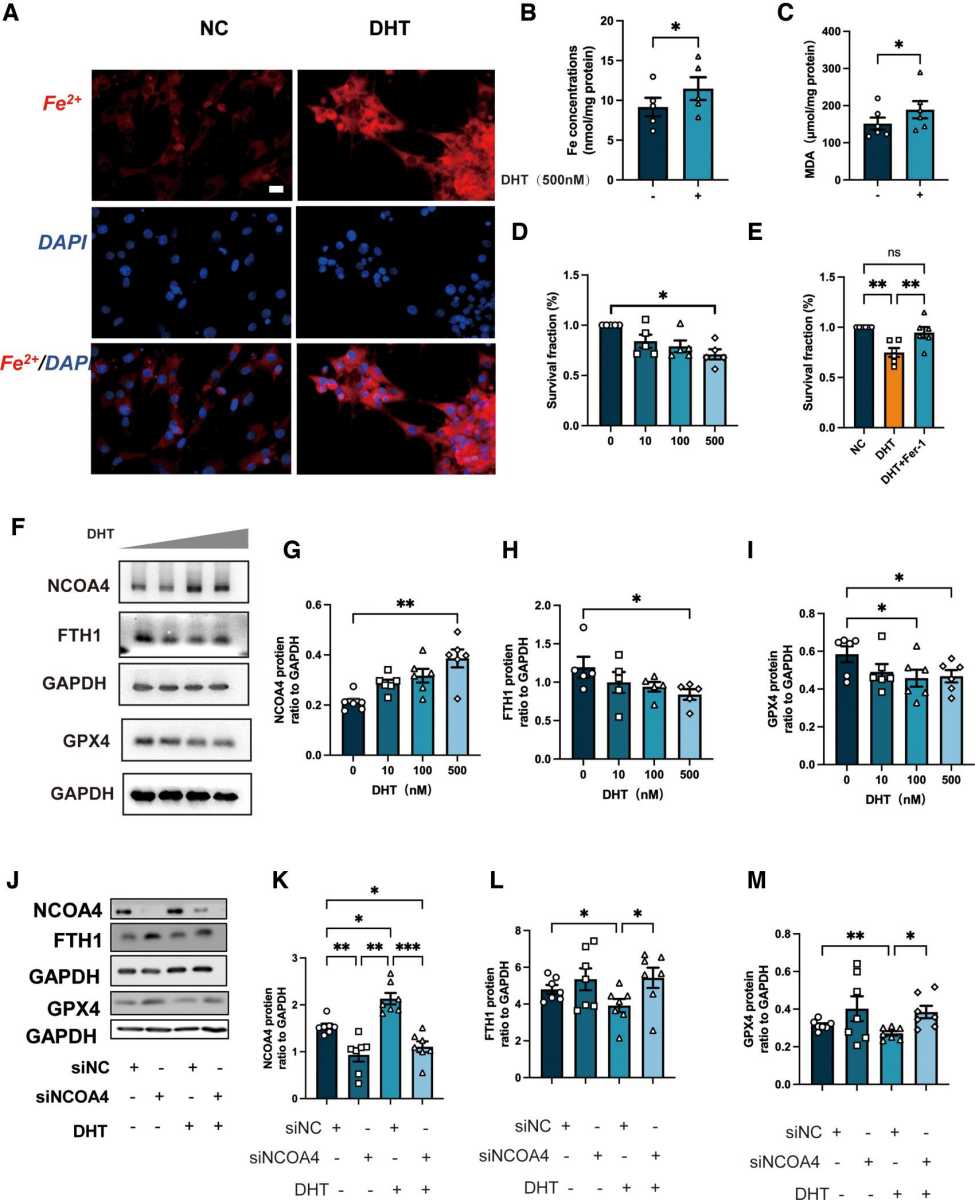
**Dihydrotestosterone induced ferroptosis in human granulosa cells.** (**A**). GCs were treated with DHT (500 nM) for 24 h and then Fe^2+^ was detected by FerroOrange staining (n = 3 per treatment). Scale bar = 20 μm. (**B** and **C**) Fe^2+^ and MDA concentration of GCs treated with DHT (500 nM) for 24h (n = 5 per treatment). (**D** and **E**) Cell viability of GCs treated with DHT (500 nM) for 48 h and incubated with or without Fer-1 (1 μM). (**F**–**I**) Protein abundance of the ferroptosis-related proteins NCOA4, FTH1, and GPX4. (**J**–**M**) After transfection with siNOCA4 and siNC, GCs were treated with or without DHT (500 nM) for 24 h, and then protein abundance of the ferroptosis-related proteins NCOA4, FTH1, GPX4 was measured. Data were analyzed using one-way ANOVA with Tukey’s multiple comparison *post-hoc* test and presented as mean ± SEM. *P < 0.05; **P < 0.01; ***P < 0.001. DHT: dihydrotestosterone; Fer-1: ferrostatin-1; FTH1: ferritin heavy chain 1; GC: granulosa cell; GPX4: glutathione peroxidase 4; MDA: malondialdehyde; NCOA4: nuclear receptor coactivator 4; siNC: control siRNA.

Previous studies have associated the hyperandrogenism of PCOS with altered programmed death of GCs in PCOS and ferroptosis in prostate cancer (Kumar *et al.*, 2021). To determine if DHT has effects on ferroptosis in GCs in PCOS, we performed intracellular Fe^2+^ staining and Fe^2+^ concentration detection in GCs treated with DHT (500 nm, 24 h) and found that Fe^2+^ levels were increased post-DHT treatment (Fig. 6A and B and Supplementary Fig. S4). In parallel, MDA concentrations were much higher in the GCs after DHT treatment (Fig. 6C). A CCK-8 test revealed that the cell viability of GCs decreased in a dose-dependent manner following DHT treatment (10 100, and 500 nM), while the ferroptosis inhibitor, Fer-1 (1 μM, 24 h), reversed the decrease (Fig. 6D and E). Notably, the abundance of the ferritinophagy core protein, NCOA4, was induced by DHT treatment in a dose-dependent manner, the level of FTH1 decreased in a dose-dependent manner after DHT treatment (10,100, and 500 nM), and the ferroptosis suppressor protein GPX4 was also reduced (Fig. 6F–I). These results indicated that ferritinophagy induced by DHT in ovarian GCs was mediated by NCOA4. To further confirm the mechanism of DHT-induced ferroptosis in ovarian GCs, we knocked down NCOA4 and found that the diminished abundance of FTH1 and NCOA4 after DHT treatment was reversed (Fig. 6J–M). These data suggested the NCOA4-dependent ferritinophagy and GPX4 axis activation are involved in DHT-induced ovarian GC ferroptosis.

### Ovarian ferroptosis induced hyperandrogenism and polycystic ovaries in rats

**Figure 7.**
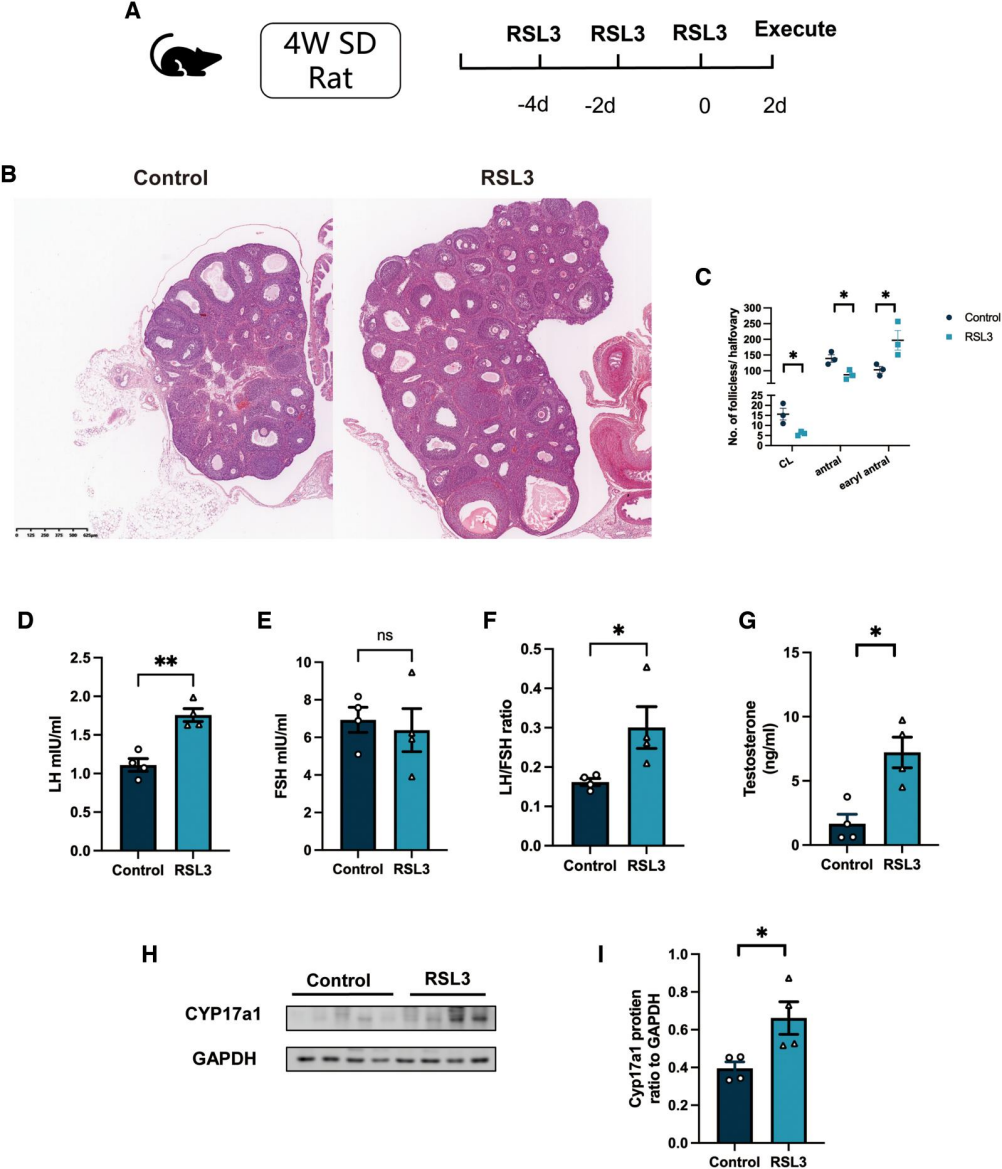
**Ferroptosis induced an increased LH/FSH ratio, hyperandrogenism, and polycystic ovaries in rats.** Rats were treated with the ferroptosis inducer RSL3 (10 μM, i.p. qod). (**A**). Diagrammatic representation of the rats from the RSL3 group treatment. (**B**), Representative histological section images of ovaries from the control and RSL3 groups. (**C**) The number of CL, antral and early antral follicles in the ovaries of rats from each group. (**D**–**G**). LH levels, FSH levels, the LH/FSH ratio, and testosterone levels of rats in the control and RSL3 groups (n = 4 per group). (**H**–**F**) The protein abundance of CYP17a1 in ovaries of rats from the two groups (n = 4 per group). Data were analyzed using the unpaired Student’s *t*-test and presented as mean ± SEM. *P < 0.05; **P < 0.01 versus the control group. CL: corpus luteum; CYP17a1: cytochrome P450 17α-hydroxylase; qod: once every other day; SD: Sprague-Dawley.

Since DHT enhanced ferroptosis in ovarian GCs, we further asked if ferroptosis in the ovary could induce hyperandrogenism as well. To answer this question, we treated the rats with the ferroptosis activator RLS3 (Fig. 7A). Surprisingly, the typical PCOS-like ovarian morphology was observed after RSL3 treatment (Fig. 7B). The number of CL was significantly decreased together with decreased numbers of antral follicles and increased early antral follicles in the ovaries of RSL3-treated rats compared to those of rats in the control group (Fig. 7C). There was no significant difference in the FSH levels between the two groups of rats; however, the LH levels and LH/FSH ratio were higher in the RSL3-treated group than in the control groups (Fig. 7D–F). Of importance, the serum testosterone concentration of rats was increased after RSL3 treatment (Fig. 7G). In females, androgen is mainly produced by the ovary. Cytochrome P450, 17α-hydroxylase (CYP17A1) is the key enzyme involved in androgen production and the etiology of PCOS (Heidarzadehpilehrood *et al.*, 2022). The abundance of CYP17A1 protein was significantly increased in the ovaries of rats treated with RSL3 (Fig. 7H and I). These results suggested that ovarian ferroptosis could induce androgen production and exacerbate PCOS development.

## Discussion

**Figure 8.**
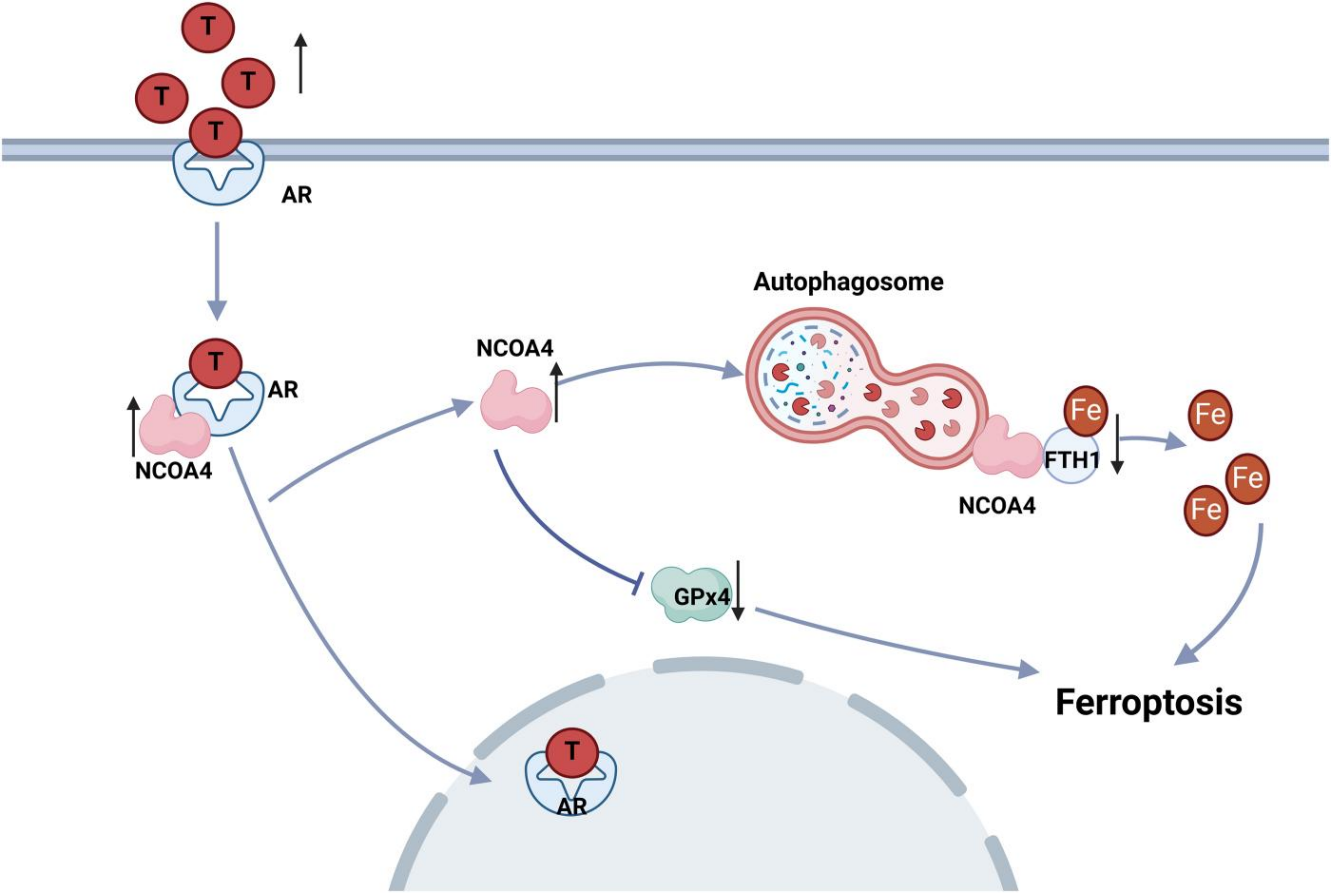
**Diagram illustrating the molecular network of this study.** Increased DHT increases NCOA4 content in ovarian granulosa cells, which may result in increased ferritinophagy, and a decrease in FTH1, which leads to an elevation of Fe^2+^ concentrations. The increased NCOA4 induces a decrease of GPX4 in the ovarian granulosa cells. These two aspects combine to enhance ferroptosis in PCOS ovaries. AR: androgen receptor; DHT: dihydrotestosterone; FTH1: ferritin heavy chain 1; GPX4: glutathione peroxidase 4; NCOA4: nuclear receptor coactivator 4; PCOS: polycystic ovary syndrome; T: testosterone.

PCOS, the most frequent endocrine disorder among women of reproductive age, is associated with a 15-fold increased risk of anovulation infertility (Joham *et al.*, 2015, 2022). Despite the high prevalence and prominent ovarian dysfunction, the underlying mechanism of PCOS development is yet to be elucidated. In this study, we demonstrated the critical involvement of NCOA4-dependent ferroptosis induced by androgen in PCOS progression. Our data demonstrated intracellular Fe^2+^ accumulation, ROS release, lipid peroxidation, mitochondrial injury, ferroptosis cargo receptor NCOA4 upregulation, and iron storage protein FTH1 and ferroptosis key suppressor GPX4 loss, which resulted in GC ferroptosis in both PCOS patients and the DHEA-induced PCOS rat model. All these changes and a series of PCOS traits, including polycystic ovarian morphology and ovulation disorders, were rescued after treatment with the ferroptosis inhibitor Fer-1 in the rat model; the mechanism of which is that DHT induces NCOA4-dependent ferritinophagy and GPX4 loss, leading to the ferroptosis in GCs (Figs 6 and 8). Remarkably, we have found a possible ‘vicious cycle’ between hyperandrogenism and ferroptosis in PCOS. Our *in vivo* study revealed that the ferroptosis inducer, RSL3, induced polycystic ovaries and that hyperandrogenism with CYP17a1 increased in rats. Taken together, we propose that ferroptosis shapes the vicious cycle with hyperandrogenism and aggravates PCOS, which identifies a ferroptosis inhibitor as a potential clinical treatment option for PCOS.

Over the past decades, understanding the types and specific pathways of cell death involved in follicular development has been an emerging topic. Multiple studies have observed increased programmed cell death (including apoptosis and autophagy) in the ovaries from women with PCOS or PCOS animal models (Matsuda *et al.*, 2012; Li *et al.*, 2019; Liu *et al.*, 2020; Wang *et al.*, 2020; Tong *et al.*, 2022; Stringer *et al.*, 2023). Similar to previous studies, our findings reveal that ferroptosis, a recently identified programmed cell death characterized by Fe^2+^-dependent phospholipid peroxidation (Dixon *et al.*, 2012; Mou *et al.*, 2019), was also excessive in ovaries from women with PCOS (Figs 1 and 2). Recent studies have revealed the aberrant expression of noncoding RNA in PCOS patients and then explored the potential effects of these noncoding RNAs on ferroptosis in immortal cell lines (Zhang *et al.*, 2021; Tan *et al.*, 2022). Abnormally expressed ferroptosis-related proteins in uterine and placental tissues in PCOS rat models have also been evaluated (Zhang *et al.*, 2020c; Hu *et al.*, 2021). No direct evidence has ever clarified the relative ferroptosis level in PCOS ovaries compared to the non-PCOS ovaries; therefore, the role of ferroptosis in the pathogenesis of PCOS and the underlying mechanism involved remained unknown. In the present study, increased Fe^2+^ and MDA concentrations were detected in GCs from PCOS patients and in ovaries obtained from PCOS rat models. At the same time, abnormal mitochondria, that appeared shorter with increased membrane intensity, as previously described in ferroptotic cell death, were also observed in both primary GC from PCOS patients and in ovaries obtained from PCOS rat models (Dixon *et al.*, 2012). To the best of our knowledge, our findings confirm those of previous studies and, for the first time, provide direct evidence of excessive ferroptosis in PCOS ovaries *in vivo* and *in vitro*.

To clarify the effect of ferroptosis in PCOS development and the potential therapeutic effect of the ferroptosis inhibitor, we treated PCOS rats with Fer-1, a type of ferroptosis inhibitor that is of benefit in multiple conditions, including tumors, ischemia/reperfusion injuries, and pre-eclampsia (Mou *et al.*, 2019; Miotto *et al.*, 2020; Zhang *et al.*, 2020b; Mahoney-Sánchez *et al.*, 2021; Yan *et al.*, 2021). However, no significant effect of Fer-1 on PCOS has been demonstrated. We observed that Fer-1 treatment ameliorates ovarian ferroptosis in PCOS (Fig. 3). Surprisingly, we found that a number of PCOS traits were alleviated, including increased weight, decreased ovarian weight, impaired glucose homeostasis, disrupted sexual hormones, hyperandrogenism, acyclicity, and morphological changes of polycystic ovaries (Fig. 4). It is known that both oligo-ovulation/anovulation and declined oocyte quality result in the subfertility in patients with PCOS (Azziz *et al.*, 2016; Li *et al.*, 2021b), while single-cell transcriptomic analysis demonstrated that altered mitochondrial dynamics could be one of the causes (Qi *et al.*, 2020). In previous studies, ferroptosis was found to be involved in the reduction of oocyte quality (Li *et al*., 2021a; Ni *et al.*, 2022). Interestingly, Fer-1 treatment protected against the decreased oocyte number and lower MII oocyte rate in the PCOS rats (Fig. 5). To the best of our knowledge, this is the first study to demonstrate that a ferroptosis inhibitor could ameliorate the clinical phenotype of PCOS and also the first study to reveal a crucial role for ovarian ferroptosis in PCOS progression. However, more safety tests (including the effects of Fer-1 in control animal and the offspring tests) are needed to support the potential translational use of Fer-1 in the treatment of PCOS. Also, the capacity for fertilization and the development of blastocysts should be explored after Fer-1 treatment. Additionally, it is important to explore the underlying mechanisms involved in the improvement of metabolism in PCOS rats after Fer-1 treatment.

Recently, ferroptosis was identified as an autophagic death and was named ferritinophagy (Gao *et al.*, 2016). This kind of autophagy depends on the cargo receptor NCOA4, to degrade ferritin and promote ferroptosis (Hou *et al.*, 2016). NOCA4, which is also known as ARA70, is a specific enhancer of AR action (Culig *et al.*, 2004). As is well known, androgen excess is one of the most important causes of PCOS and it exaggerates the programmed cell death in the ovary according to the previous studies (Wang *et al.*, 2020; Zeng *et al.*, 2023). Considering the vital role of NCOA4 in the androgen signaling pathway, we assume ferritinophagy may, at least in part, participate in the increased GC ferroptosis of PCOS. In the present study, an increased abundance of NCOA4 and the reduced abundance of FTH1 and the ferroptosis suppressor GPX4 were observed in ovarian GCs from PCOS patients and in the ovaries of rats with DHEA-induced PCOS (Figs 1 and 2). We then used primary ovarian GCs to explore the effects of DHT on ferroptosis. After treating human GCs with DHT *in vitro*, GCs were found to have increased Fe^2+^ and MDA concentrations, and Fer-1 treatment reversed the decreased cell viability of GCs after DHT treatment. The ferritinophagy-related proteins (NCOA4 and FTH1) were changed in a dose-dependent manner, and the abundance of GPX4 decreased as well. In the current study, an *in vitro* siRNA-knockdown experiment confirmed the involvement of NCOA4-dependent ferritinophagy in DHT-induced ferroptosis. The *in vitro* siRNA-knockdown experiment also revealed that GPX4 reduction induced by DHT was dependent on NCOA4 as well (Fig. 6). These findings implied that DHT induces NCOA4-dependent ferritinophagy in GCs from individuals with PCOS (Fig. 8). However, it is noted that the levels of ferroptosis-related protein (especially FTH1) were not decreased consistently in patients with PCOS. Since PCOS is a complex disease with high heterogeneity, whether the level and the mechanism of ferroptosis varied in different subtypes of women with PCOS needs to be explored in the future. Furthermore, *in vivo* experiments, such as those involving GCs-specific NCOA4 knockout mice, are needed to explore the roles played by NCOA4 in DHT-induced ferroptosis and in the development of PCOS. Taken together, here, we have presented both *in vivo* and *in vitro* results as the first evidence for the crucial role of hyperandrogenism in ovarian ferroptosis in PCOS. To the best of our knowledge, this study revealed the underlying mechanism of DHT-induced ferroptosis in ovarian GCs for the first time.

To reconfirm the role of ovarian ferroptosis in PCOS progression, we treated rats with the ferroptosis activator RSL3. Surprisingly, these rats showed some PCOS-like traits including polycystic ovarian morphology, LH hypersecretion in combination with an increased LH/FSH ratio, and hyperandrogenism post-treatment. The increase in number of early antral follicles and the reduction in number of CLs and antral follicles indicated the vital effect of ferroptosis in GCs on follicular development, just like other types of cell death (Yadav *et al.*, 2018). Ovarian androgen hypersecretion is the main source of excess androgen in PCOS; theca cell defects were found to account for this phenomenon (Rosenfield and Ehrmann, 2016). CYP17A1 is the rate-limiting enzyme in the process of androgen production. We have detected an augmented abundance of CYP17A1 in the ovaries of RSL3-treated rats (Rosenfield and Ehrmann, 2016). Based on these findings, we reasoned that there might be a “feed-forward” loop between ovarian ferroptosis and androgen excess as we conducted i.p. injection of RLS3 into the rats. An impact of RSL3 on the other organs cannot be excluded and relevant mechanisms should be further explored in future studies to confirm the vicious circle of exaggerated ovarian ferroptosis and PCOS progression.

## Conclusion

Our findings support a likely role for ovarian ferroptosis in the pathogenesis of PCOS. First, the inhibition of ovarian ferroptosis by Fer-1 can ameliorate a cluster of PCOS traits including hyperandrogenism and ovulatory dysfunction. Second, NCOA4-dependent ferritinophagy and GPX4 axis activation may (at least partially) contribute to the DHT-induced ferroptosis in ovarian GCs. Third, there could be a vicious cycle between ovarian ferroptosis and the development of PCOS. We believe this study provides vital clues for potential therapeutic strategies for patients with PCOS in the future by regulating iron metabolism and lipid peroxidation.

## Supplementary Material

hoae013_Supplementary_Data

## Data Availability

The datasets generated during and/or analyzed during the current study are available from the corresponding author upon reasonable request.
