# Correction to: Ovarian ferroptosis induced by androgen is involved in pathogenesis of PCOS

**DOI:** 10.1093/hropen/hoaf078

**Published:** 2025-12-22

This is a correction to Xinyu Li, Yunying Lin, Xiaoyue Cheng, Guangxin Yao, Jufang Yao, Shuanggang Hu, Qinling Zhu, Yuan Wang, Ying Ding, Yao Lu, Jia Qi, Hanting Zhao, Xuejiao Bian, Yanzhi Du, Kang Sun, Hugo Vankelecom, Yun Sun, Ovarian ferroptosis induced by androgen is involved in pathogenesis of PCOS, *Human Reproduction Open*, Volume 2024, Issue 2, hoae013, https://doi.org/10.1093/hropen/hoae013.

In Figure 1 of this article, the NCOA4, FTH1 and both GAPDH blots shown in Fig. 2C and D were erroneously duplicated in Fig. 1E and F. The correct versions of these blots were provided for Figure 1 in the original version of the manuscript submitted to the journal, with the duplication errors inadvertently introduced during figure reassembly for the revised manuscript. The authors have provided the original raw data and a new Figure 1, shown below, which includes the correct blots originally submitted for Figure 1.

**Figure 1.**
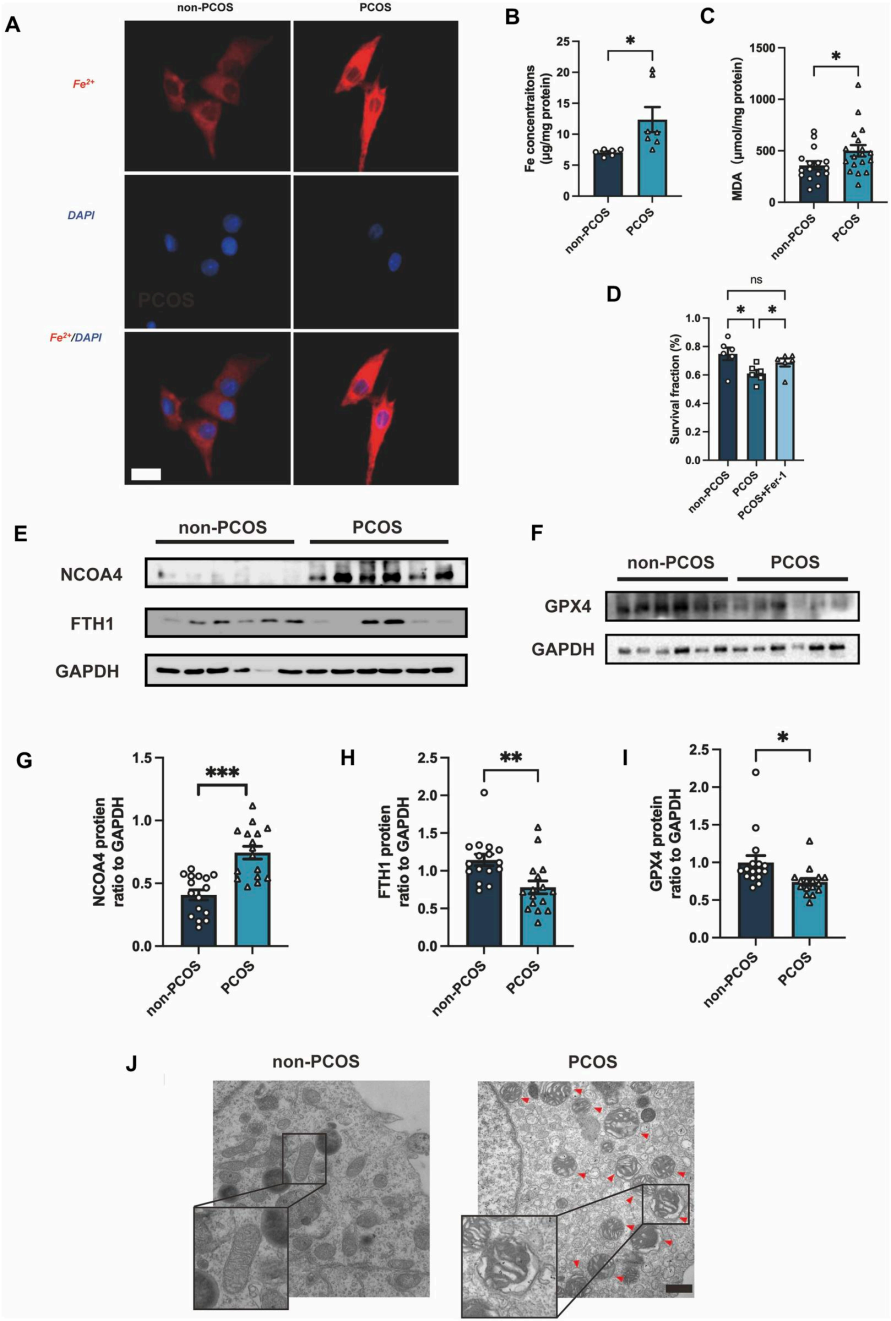
**Elevated ferroptosis in granulosa cells from patients with PCOS**. (**A**). Representative images of intracellular Fe^2+^ in GCs from PCOS patients and non-PCOS patients detected by FerroOrange (n = 3 per group). Scale bar = 20 μm. (**B** and **C**) The cellular Fe^2+^ concentration (n = 6–7 per group) and MDA concentration (n = 16–18 per group) of GCs from non-PCOS and PCOS groups were also detected. (**D**) GCs isolated from PCOS patients were treated with Fer-1 (1 μM) for 48 h and then cell viability was detected by CCK-8 (n = 6 per group). (**E**–**I**) Protein abundance of ferroptosis-related proteins NCOA4, FTH1, GPX4 in GCs from PCOS patients and non-PCOS patients (n = 16 per group), assessed by western blotting. (**J**) Representative TEM images of GCs from PCOS patients and non-PCOS patients. Scale bar = 500 nm. Red arrowheads indicate abnormal mitochondria. Data were analyzed using unpaired Student’s *t*-test and presented as mean ± SEM. *P < 0.05; **P < 0.01, ***P < 0.001, ns: no significance versus the non-PCOS group. CCK-8: cell counting kit-8; Fer-1: ferrostatin-1; FTH1: ferritin heavy chain 1; GCs: granulosa cells; GPX4: glutathione peroxidase 4; MDA: malondialdehyde; NCOA4: nuclear receptor coactivator 4; PCOS: polycystic ovary syndrome; TEM: transmission electron microscopy.

This correction does not affect the Results, Discussion and conclusions presented in the article. These details have been corrected only in this correction notice to preserve the published version of record.

