# Supplementary material for: Ovarian ferroptosis induced by androgen is involved in pathogenesis of PCOS
Source: Hum Reprod Open. 2024 Feb 28;2024(2):hoae013. doi: 10.1093/hropen/hoae013 (PMC10973940; doi:10.1093/hropen/hoae013)
Supplement: hoae013_Supplementary_Data [file hoae013_supplementary_data.pdf]

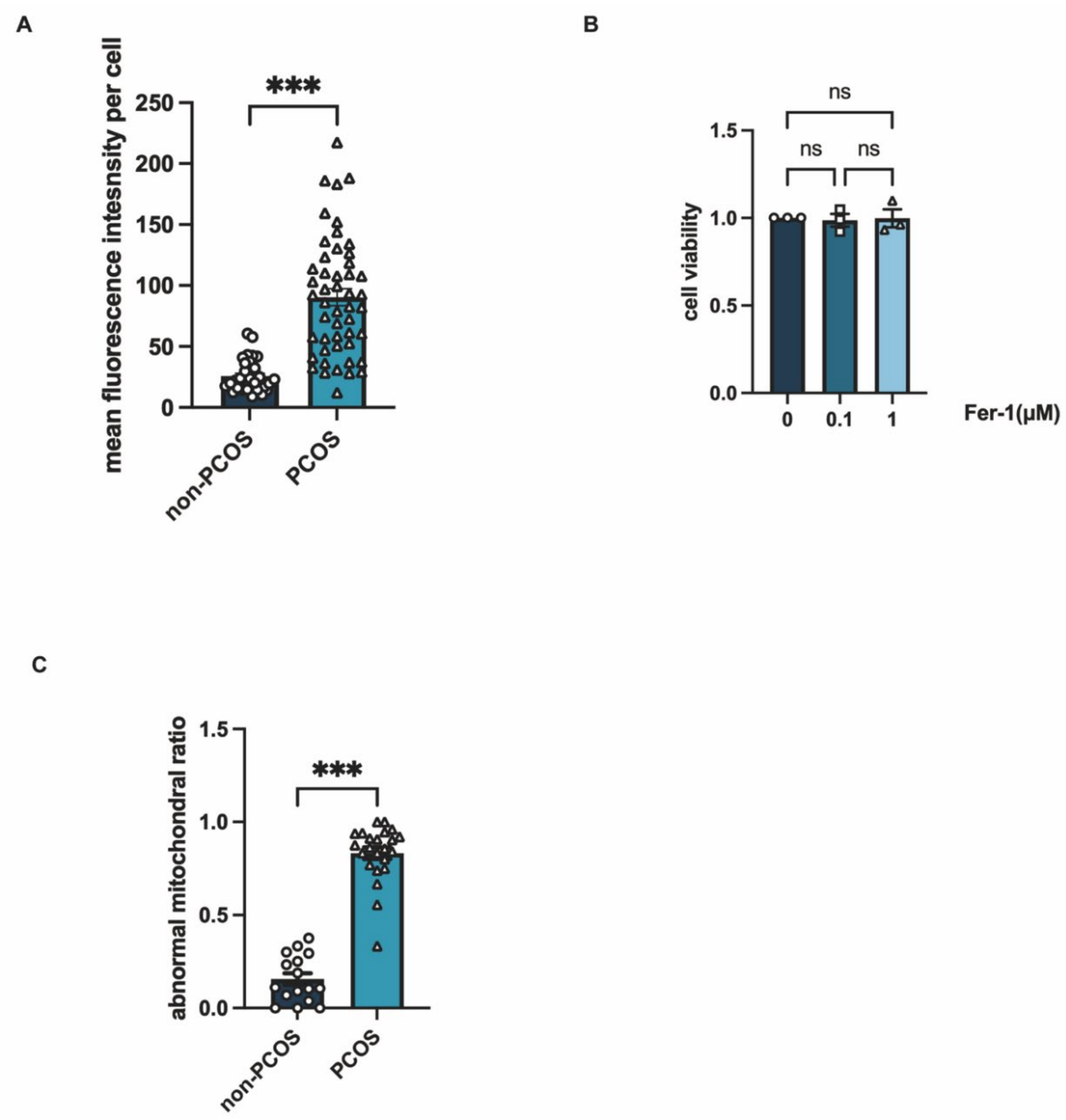

Supplementary Figure S1. Elevated Fe^{2+} and abnormal mitochondrial ratio.

(A). Mean fluorescence intensity per cell of intracellular Fe^{2+} staining in GCs from PCOS patients and the non-PCOS patients detecting by FerroOrange (n=3 per group). (B). The GCs from non-PCOS patients were treated with Fer-1 (0.1, 1 μM) for 48 h and then cell viability was detected by CCK-8 (n=3 per group). (C). The abnormal mitochondrion ratio in GCs from patients with or without PCOS. Data were analysed using unpaired Student's *t*-test and presented as mean ± SEM. *** $P < 0.001$, ns means no significance vs the non-PCOS group. GC: granulosa cell, CCK-8: cell counting kit-8.

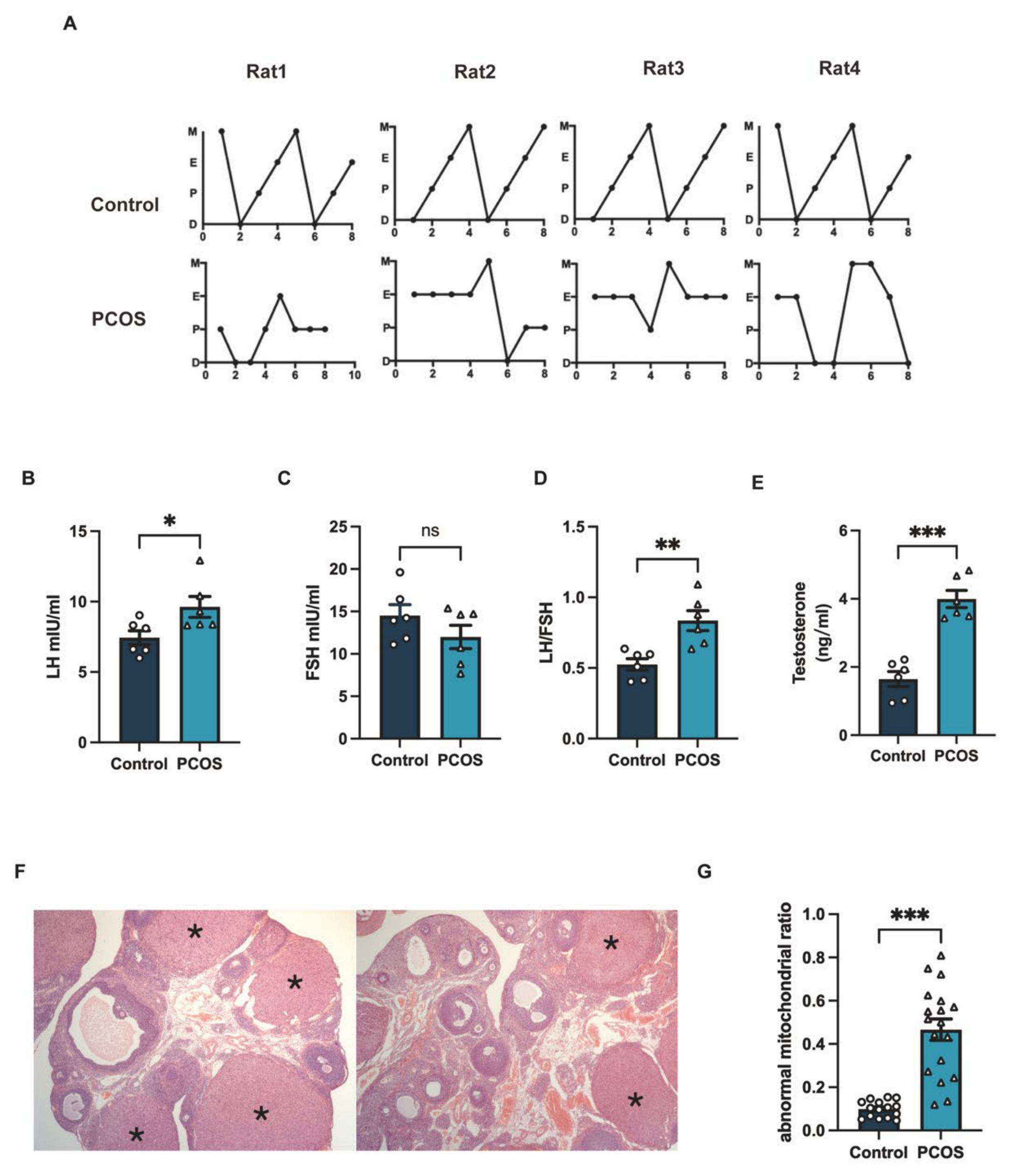

Supplementary Figure S2. Establishment of PCOS rat model.

(A). The representative estrous cycle of rats in 8 days from the control group and the PCOS group. D, diestrus; E, estrus; M, metestrus; P, proestrus. (B-E). LH levels, FSH levels, the LH/FSH ratio, and the T levels of rats in the Control, PCOS group (n=8 per group). (F). Representative histological section images of ovaries from the Control group and the PCOS group. Asterisks indicate corpus luteum. (G) The abnormal mitochondrion ratio in GCs from

rats in the control group and the PCOS group. Data were analysed using unpaired Student's *t*-test and presented as mean ± SEM. *** $P < 0.001$, ns means no significance vs the control group. PCOS: polycystic ovary syndrome, FSH: follicle stimulating hormone, LH: luteinizing hormone.

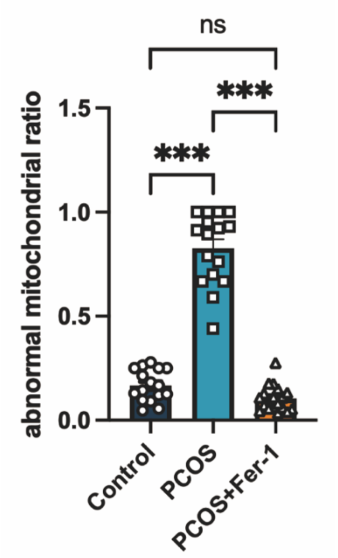

Supplementary Figure S3. Comparison of the abnormal mitochondrion ratio in GCs from rats in the control group, the PCOS group, and the PCOS+Fer-1 group.

Data were analysed using one-way ANOVA with Tukey's multiple comparison posthoc test and presented as mean ± SEM. *** $P < 0.001$, ns means no significance vs the control group. GC: granulosa cell, Fer-1: ferrostatin-1.

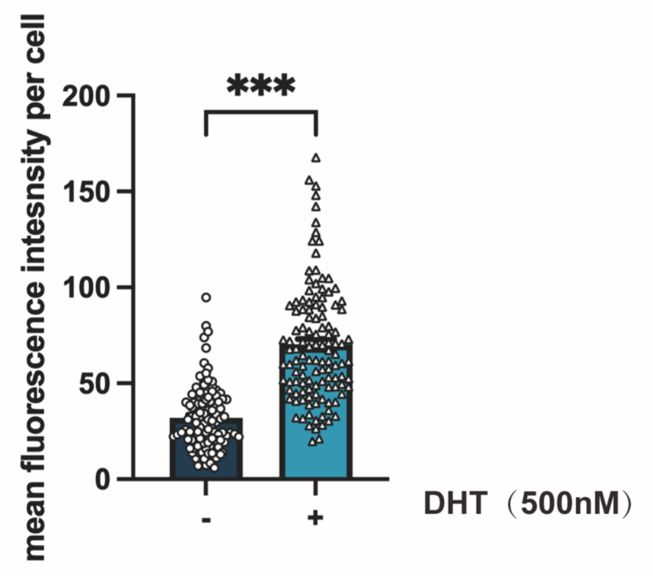

Supplementary Figure S4. Mean fluorescence intensity per cell of intracellular Fe^{2+} staining in GCs treated with DHT (500nM) for 24h detected by FerroOrange (n=3 per treatment). Data were analysed using unpaired Student's *t*-test and presented as mean ± SEM. *** $P < 0.001$. GC: granulosa cell, DHT: dihydrotestosterone.

Supplementary Table S1. Demographic features and clinical outcomes of recruited patients.

	Non-PCOS(n=16)	PCOS(n=16)	p Value
Age (years)	30.31 ±0.8048	29.50 ± 0.8563	0.4946
BMI (kg/m^2)	21.69 ± 0.5912	22.47 ± 0.7119	0.4037
Basal FSH (mIU/mL)	6.463 ± 0.2698	5.844 ± 0.3708	0.1934
Basal LH (mIU/mL)	5.348 ± 0.4641	9.905 ± 1.765	0.0183
LH/FSH ratio	0.8334 ± 0.06492	1.65 ± 0.2449	0.0031
Basl T (nmol/L)	0.5629 ± 0.04881	1.464 ± 0.1418	<0.0001
AMH (ng/mL)	4.084 ± 0.41769	10.61 ± 1.65	0.0006
No. of oocytes retrieved	17.00 ± 1.140	24.06 ± 2.552	0.0170
MII oocyte rate	0.9023 ± 0.02462	0.8350 ± 0.03101	0.0995

Data were analysed using unpaired Student’s t-test and presented as mean ± SEM. $P < 0.05$ is considered significant. T: testosterone; AMH: anti-Müllerian hormone
